# Effect of pioglitazone therapy on high sensitive C-reactive protein and lipid profile in diabetic patients with renal transplantation; a randomize clinical trial

**DOI:** 10.12860/jnp.2015.10

**Published:** 2015-04-01

**Authors:** Rana Arashnia, Kobra Roohi-Gilani, Hamidreza Karimi-Sari, Niloofar Nikjoo, Ali Bahramifar

**Affiliations:** ^1^Urology and Nephrology Research Center, Shahid Labbafinejad Medical Center, Shahid-Beheshti University of Medical Sciences, Tehran, Iran; ^2^Students’ Research Committee, Baqiyatallah University of Medical Sciences, Tehran, Iran; ^3^Students’ Research Committee, Iran University of Medical Sciences, Tehran, Iran

**Keywords:** Pioglitazone, C-reactive protein, Cholesterol, Diabetes mellitus, Kidney transplantation

## Abstract

*Background:* Inflammation has a major role in disease lead to renal failure and diabetes mellitus, controlling inflammation in diabetic kidney receivers could decrease morbidity and mortality.

*Objectives:* This study designed for evaluating the efficacy of pioglitazone on C-reactive protein and lipid profile in diabetic kidney transplant receivers.

*Patients and Methods:* In this double blinded clinical trial, 58 diabetic renal transplant receivers, in first month after transplantation, randomized into two groups; receiving insulin and pioglitazone (15 mg tablet daily, group A); and insulin and placebo (group B). Blood pressure, weight, body mass index (BMI) and laboratory data compared in before and after 4-month treatment in two groups by SPSS.

*Results:* Fifty-eight patients with mean age of 44.15 ± 2 years included. There were no significant difference between groups in demographic data and other baseline measured variables (*P* > 0.05) .The mean weigh and BMI were slightly increased in group A and decreased in group B. The mean hs-CRP was decreased 4.82 mg/dL in group A and 1.93 mg/dL in group B (*P* = 0.007). The mean total serum cholesterol was significantly decreased 34 mg/dL in group A and 18.07 mg/dL in group B (*P* = 0.027). The mean serum HDL-C was significantly increased 13.31 mg/dL in group A and 5.89 mg/dl in group B (*P* < 0.001).

*Conclusions:* Pioglitazone seems to be a safe drug for reducing serum lipids and CRP in kidney transplant receivers with diabetes mellitus in short term. Long term effect of this drug could be evaluated in future studies.

**Implication for health policy/practice/research/medical education:**

Pioglitazone could control inflammatory process in diabetic renal transplant receivers and decrease inflammatory related morbidities.

## 1. Background

Kidney transplantation is the most effective treatment for chronic renal failure. Patients with kidney transplantation are forced to use glucocorticoids in order to prevent rejection, which is the most basic element of transplantation protocols (1). Treatment with glucocorticoids is associated with increased risk of diabetes mellitus, insulin resistance, weight gain and blood pressure. Other factors such as hepatitis-C virus (HCV) or cytomegalovirus (CMV) infections can cause blood glucose disorder in patients with kidney transplantation. These cases can cause conflicts of islets of Langerhans cells. Asymptomatic CMV infection increases the risk of diabetes after renal transplantation (2,3). Diabetes is the most common cause of chronic renal failure and in some cases; diabetes mellitus occurs immediately after transplantation. One of the effects of uncontrolled blood glucose is increasing inflammatory processes that have negative impacts on the prognosis of renal transplantation (1-3). In conducted studies, using Thiazolidinedione has been emphasized to reduce adverse effects of uncontrolled blood glucose that one of these effects is reducing the inflammatory processes (4).

Controlling inflammatory processes in diabetic patients with kidney transplantation will have dramatic effects on reducing mortality, because the inflammatory processes are integral component of the diseases leading to renal failure and diabetes. Also, the experimental data showed that inflammation plays a role in atherogenesis process (5).

These drugs increase the sensitivity of insulin receptors by PPAR-Ɣ receptors stimulation and reduce insulin resistance (6). Pioglitazone is a drug of thiazolidinedione category that in addition to controlling glucose, reduces the inflammatory processes, and reduces the inflammatory processes. Based on studies, pioglitazone does not interfere with common transplant medications such as cyclosporine and mycophenolate mofetil. These drugs have been identified secure for damages to the connective tissue (4,5). Pioglitazone consumption is not contraindicated with mentioned medications and it does not cause any change in the creatinine or tacrolimus levels. Pioglitazone does not increase creatinine or the need for immunosuppressive drugs in patients with kidney transplantation (7-10).

## 2. Objectives

This study designed to investigate the effects of pioglitazone on inflammatory processes in diabetic patients with kidney transplantation and if this medicine would be effective, it will help to increase the quality of life, duration of life and kidney function, reduce health care costs related to transplantation, graft rejection, and the need of alternative treatment in patients with kidney transplantation.

## 3. Patients and Methods

### 3.1. Study patients

**Figure 1:**
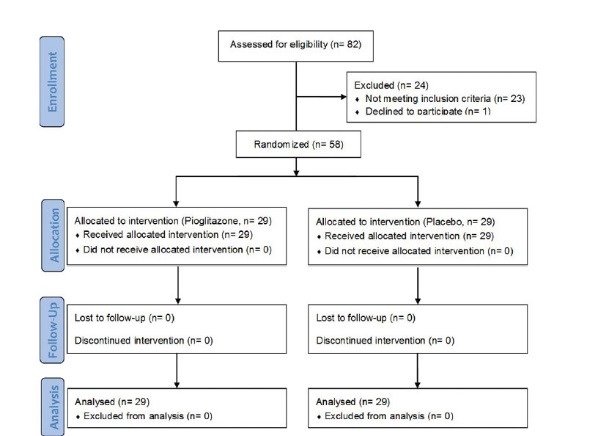
Study flow chart according to CONSORT 2010

In this double blind randomized clinical trial, patients who had undergone kidney transplantation with diabetes, entered to the study. Fasting blood glucose (FBS) more than 126 mg/dl, from America diabetes association diagnostic criteria, was used to diagnose diabetes. The study protocol was designed in compliance with the principles of the Helsinki Convention. Cigarette smokers or patients with heart failure FC II, III, hepatitis B and C, GFR less than 30 cc/min, pregnancy, acute rejection with progressive renal impairment and creatinine more than 1 mg/dl did not enter to the study. Patients gave written consent before entering the study. Patients divided randomly into two groups by closed label method. First group received insulin therapy with daily one 15 mg pioglitazone tablet (A) for four months and second group received insulin therapy with placebo tablet (B). Placebo tablets were produced identical in shape and size with pioglitazone by its manufacturer and drug prescriber, those who visit the patients and filled the checklists in all stages as well as the person who performed the analysis were unaware of drug compounds of both groups. At the beginning of the study, all patients were examined and interviewed, information about various diseases and history of present illness and consumed drugs were assessed through biography and present documents. On height, weight and waist circumference examinations, heart, lung and lower extremity examinations carried out for presence of congestive heart failure and other mentioned diseases in the exclusion criteria (Figure 1).

### 3.2. Laboratory assessments

Patients’ blood samples were taken to check for uric acid, lipid profile and hs-CRP. All patients were visited monthly, their blood glucose and creatinine were controlled monthly by experiments and if any problems or side effects were seen, patients were excluded from study. After four months the examinations were performed again.

Initial and four months laboratory tests were conducted using Pars Azmoon lab kits (TG91008, CHL91012, CRP92002, LDL-C92004, HDL-C92002, and Glu92008) at Labbafinejad hospital laboratory.

### 3.3. Ethical issues

(a) The research followed the tenets of the Declaration of Helsinki; (b) informed consent was obtained; and (c) the research was approved by the Urology and Nephrology Research Center and ethics committee of Shahid-Beheshti University of Medical Sciences and also has Iranian registry of clinical trials code of IRCT2014050117413N2.

### 3.4. Statistical analysis

Data were analyzed by SPSS version 21 (SPSS Inc. Chicago, IL) for windows. At first, study patients described using mean descriptive statistics, standard deviation, and frequency then two groups compared before intervention by independent sample *t* test and its nonparametric equivalent (Mann-Whitney) for quantitative variables, then Chi^2^ and Fisher exact tests used for qualitative variables. After treatment, groups wrer compared with above tests. Then before and after treatment amounts were compared in each group with paired sample *t* test, its nonparametric equivalent (Wilcoxon), and McNemar test. A, P amount less than 0.05 considered as significant value.

## 4. Results

A total of 58 patients were studied (29 patients in each group). Sixty-nine percent of group A were male and 31% were female. This distribution was 62 males and 38% female in group B (*P* = 0.58).

The mean age was 44.15 ± 13.82 years that was 44.41 ± 14.24 years in group A and 43.68 ± 13.67 years in group B (*P* = 0.89). The mean weight was 78.73 ± 11.39 kg and the mean BMI was 28.32 ± 3.76 kg/m2 (Table 1).

**Table 1:** Comparison of characteristics and laboratory findings before and after treatment between groups

**Variables**	**Pioglitazone (n=29)**	**Placebo (n=29)**	*** P *** **value**
Age, year	44.41 (14.24)	43.36 (13.67)	0.89
Male, frequency (%)	20 (69.0%)	18 (62.1%)	0.58
Weight, kg			
Before	78.58 (13.12)	77.87 (9.58)	0.923
After	80.67 (13.47)	77.43 (10.21)	0.306
Changes	2.09 (1.8)	- 1.45 (2.21)	<0.001
BMI, kg/m^2^			
Before	28.2 (4.06)	28.42 (3.84)	0.826
After	28.95 (4.13)	27.88 (3.53)	0.294
Changes	0.74 (0.65)	- 0.55 (0.84)	<0.001
Waste, cm			
Before	105.41 (13.25)	106.84 (10.26)	0.733
After	103.59 (12.28)	105.2 (10.96)	0.598
Changes	- 1.83 (4.01)	- 1.28 (3.82)	0.594
FBS, mg/dL			
Before	137.43 (28.81)	141.65 (67.64)	0.762
After	103.33 (21.95)	117.82 (40.28)	0.104
Changes	- 34.56 (15.97)	- 23.83 (37.65)	0.176
Uric Acid, mg/dL			
Before	5.83 (1.03)	5.62 (1.13)	0.499
After	4.87 (0.88)	4.86 (0.92)	0.951
Changes	- 0.96 (0.73)	- 0.77 (0.61)	0.317
hs-CRP, mg/dL			
Before	8.38 (7.63)	9.06 (7.96)	0.743
After	3.56 (3.18)	7.13 (7.54)	0.026
Changes	- 4.82 (4.79)	- 1.93 (2.56)	0.007
TG, mg/dL			
Before	207.34 (61.43)	196.82 (50.86)	0.481
After	158.76 (36.73)	163.31 (42.42)	0.664
Changes	- 48.59 (44.62)	- 33.52 (31.57)	0.143
Cholesterol, mg/dL			
Before	189.07 (46.04)	183.68 (45.34)	0.656
After	155.07 (33.73)	165.62 (38.65)	0.273
Changes	- 34.0 (27.78)	- 18.07 (25.59)	0.027
LDL-C, mg/dL			
Before	113.28 (25.54)	111.72 (29.76)	0.832
After	87.86 (11.38)	93.38 (17.89)	0.167
Changes	- 25.41 (20.37)	- 18.34 (18.18)	0.169
HDL-C, mg/dL			
Before	48.89 (8.34)	50.62 (8.11)	0.428
After	62.21 (9.54)	56.52 (7.65)	0.015
Changes	13.31 (7.84)	5.89 (6.26)	<0.001

**Figure 2:**
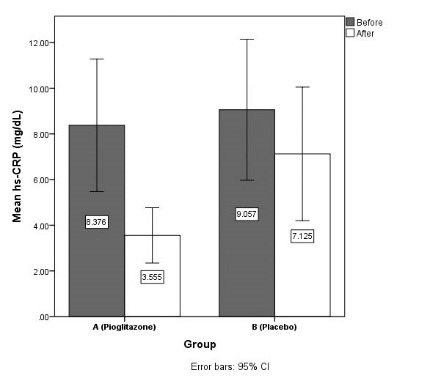
Mean hs-CRP before and after intervention in two groups.

The mean body weight was increased 2.09 ± 1.8 kg in group A and decreased 1.45 ± 2.21 kg in group B (*P* < 0.001). The mean waste circle size was decreased 1.83 ± 4.01 cm in group A and 1.28 ± 3.82 cm in group B (*P* = 0.594). The mean fasting blood sugar was decreased 34.56 ± 15.97 mg/dL in group A and 23.83 ± 37.65 mg/dL in group B (*P* = 0.176). The mean uric acid was decreased 0.96 ± 0.73 in group A and 0.77 ± 0.61 in group B (*P* = 0.317). The mean serum hs-CRP was decreased 4.82 ± 4.79 mg/dL in group A and 1.93 ± 2.56 mg/dL in group B (*P* = 0.007). In before-after evaluations (within groups), there were significant difference between before and after amounts in both groups (*P* < 0.05) (Figure 2).

### 4.1. Lipid profile

**Figure 3:**
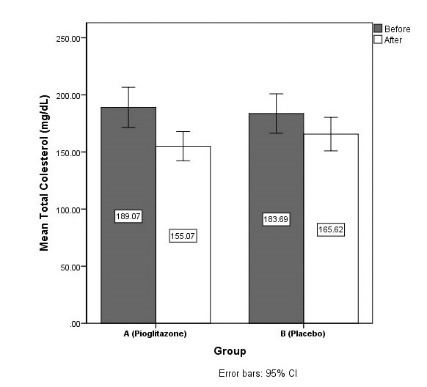
Mean total cholesterol before and after intervention in two groups

**Figure 4:**
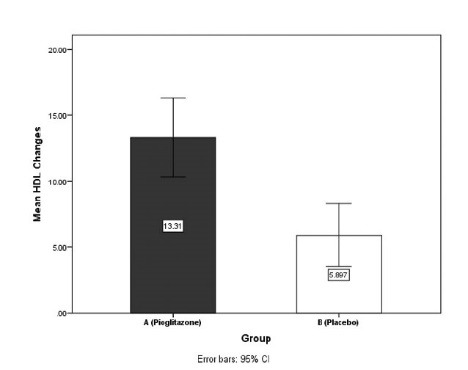
Comparison of mean HDL-C changes after intervention between pioglitazone and placebo groups

The mean triglyceride was decreased 48.59 ± 44.62 mg/dL in group A and 33.52 ± 31.57 mg/dL in group B (*P* = 0.143). Mean cholesterol was also decreased 34 ± 27.78 mg/dL in group A and 18.08 ± 25.59 mg/ dL in group B (*P* = 0.027). The mean LDL-C was decreased 25.41 ± 20.37 mg/dL in group A and 18.34 ± 18.18 mg/dL in group B (*P* = 0.169). The mean HDL-C was increased 13.13 ± 7.48 mg/dL in group A and 5.89 ± 6.26 mg/dL in group B (*P* < 0.001) (Figure 3 and 4).

In before-after evaluations (within groups), there were significant difference between before and after lipid profile amounts in both groups (*P* < 0.05).

## 5. Discussion

According to results, 15 mg pioglitazone daily added to routine insulin therapy, could decrease serum hs- CRP and cholesterol and increase serum HDL-C in diabetic renal transplant receivers. Pioglitazone could also help to control the blood glucose better.

Given that the patients enrolled to the study after renal transplantation and were treated with prednisolone and immunosuppressive drugs, it seems that these drugs have an impact on the amount of lipid reduction in both groups.

The results of this study are similar to the results of Luther et al in 2004 on serum lipids changes, however both studies did not report significant changes in lipids levels (7).

The results of the present study are inconsistent with Mattoo et al study in 2005, on significant reduction in levels of LDL-C, cholesterol, triglycerides and hs- CRP after treatment with pioglitazone. The difference is that, the target group of Mattoo’s study was not patients with kidney transplantation and significant change has not been stated in the amount of cholesterol and triglycerides in the control group. Follow-up period of patients in Mattoo’s study was six months (8). The results of the present study are consistent with the study of Hanefeld et al too (9). Likewise, the results of our study are similar to the study by Szapary et al on reduction levels of inflammatory markers and serum lipids (11). Additionally, study of Davidson et al on increase of weight and BMI and decrease of hs-CRP in pioglitazone group was in accord with the findings of our study. However, the difference is that, Davidson et al study, was not on patients with kidney transplantation which follow-up period was 24 weeks and also glimepiride drug prescribed for control group (12). Furthermore the findings of Han et al showed the changes in hs-CRP, and cholesterol in patients who taking pioglitazone, which was similar to our findings (13).

Likewise, the results of the present study was consistent with Liu et al study in 2013 on reduction of hs-CRP levels. However, the difference is that, drug taking period was 24 weeks, the control group received sitagliptin drug and they concluded that pioglitazone had a significant effect in reducing plasma glucose, hs-CRP and HbA1c (14).

Additionally, present study approved the results of the study by Suzuki et al., in 2013 on hs-CRP reduction. Importantly, blood pressure, HbA1c and fasting plasma glucose reduction are mentioned in their patients. The study stated positive effects of medication with candesartan and pioglitazone on reducing blood pressure, inflammatory markers and improving metabolism in patients with type II diabetes (10).

Due to the significant reduction in serum lipids, inflammatory markers, uric acid and fasting blood glucose of patients with kidney transplantation after administration of pioglitazone, this drug can prevent metabolic disorders after renal transplantation, however decision making about long-term effects of drug on patients with kidney transplantation needs more studies (12-15). It is recommended that in future studies, longer treatment duration be considered for patients and patients are examined for longer duration. Also, the effect of pioglitazone drug accompaniment with other anti-diabetic medications should be evaluated. The effect of this drug on blood pressure and HbA1c after transplantation could also be examined in future studies. Therefore, it is also suggested that the effect of this drug be compared with other drugs in diabetic and non-diabetic patients who received organ transplantation.

## 6. Conclusions

Oral pioglitazone seems to be a safe drug for reducing serum lipids and hs-CRP in kidney transplant receivers with diabetes mellitus in short-term evaluation. Long-term effect of pioglitazone therapy in diabetic kidney transplanted patients could be evaluated in future studies.

## Authors’ contributions

Collecting data: RA, NN, and KRG. Statistical analysis: HKS. Drafting manuscript: HKS, RA, NN, and AB. Study supervision: KRG.

## Conflict of interests

The authors declared no competing interests.

## Funding/Support

This study was extracted from residential thesis of Dr Arashnia. This study was supported by a grant from Shahid-Beheshti University of Medical Sciences (grant#135).
